# Supplementary figures and images for: Optimization of DNA extraction from human urinary samples for mycobiome community profiling
Source: PLoS One. 2019 Apr 25;14(4):e0210306. doi: 10.1371/journal.pone.0210306 (PMC6483181; doi:10.1371/journal.pone.0210306)

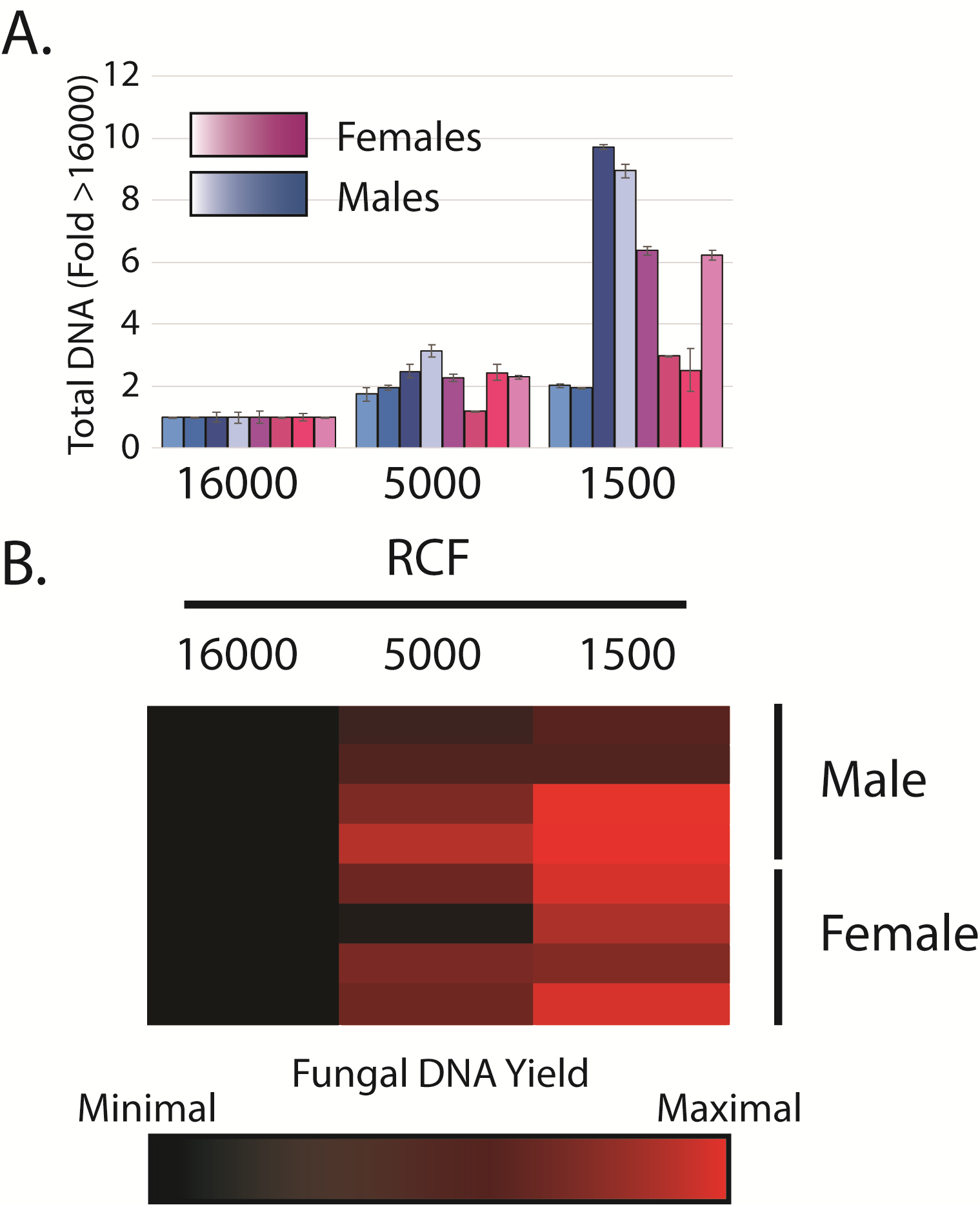

Supplement: S1 Fig — In individual subjects, lower centrifugation speeds are associated with increased total and fungal DNA yields from urine samples. Urine samples from eight subjects, four male (in blue) and four female (in pink), were divided into three 30 ml samples and subjected to centrifugation at the indicated speeds. (A) Total DNA yields from each condition were measured using a Nanodrop Spectrophotometer and expressed at a fold increase over the levels seen in the 16000 rcf sample. (B) Fungal DNA yields from these variations in centrifugation speed were calculated by quantitative PCR using primers specific for the 18S ribosomal DNA locus, then scaled across all samples. Values are expressed as a heat map, with bright red signifying the highest yields and black the lowest yields across all samples. (TIF) [file pone.0210306.s001.tif]

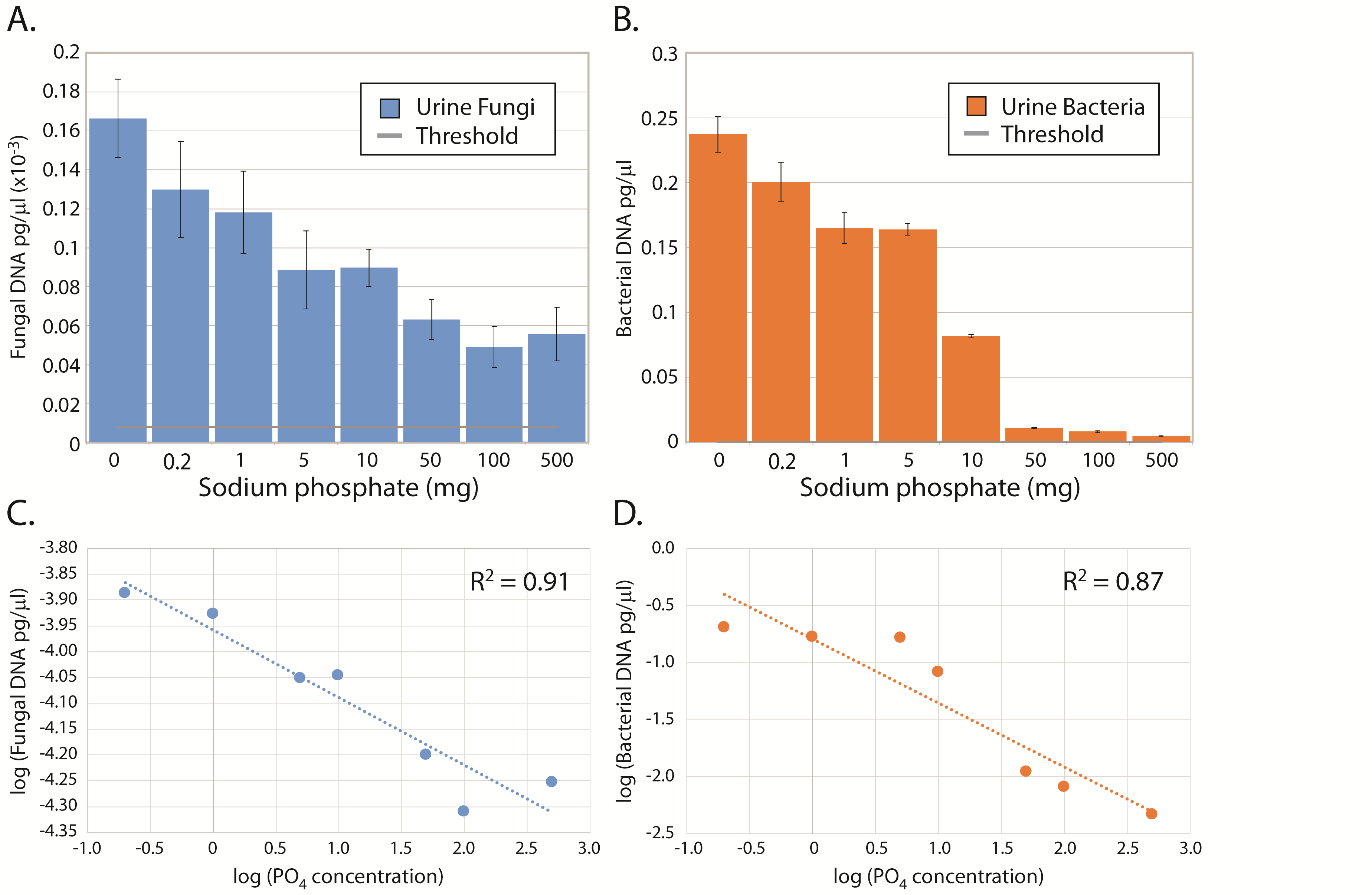

Supplement: S2 Fig — Large volume urine samples were divided into eight 30 ml samples. After adding the indicated amount of sodium phosphate to each sample, each specimen was processed according to the optimal DNA isolation protocol. (A) Fungal and (B) bacterial DNA yields from these samples were calculated by quantitative PCR in triplicate. Profound decreases in microbial DNA yields, despite equal starting DNA quantities, occur with increasing phosphate concentrations. This decrease is directly dependent on phosphate concentration, as seen in plots of the log of the phosphate concentration against the log of the fungal (C) and bacterial (D) DNA yields. (TIF) [file pone.0210306.s002.tif]

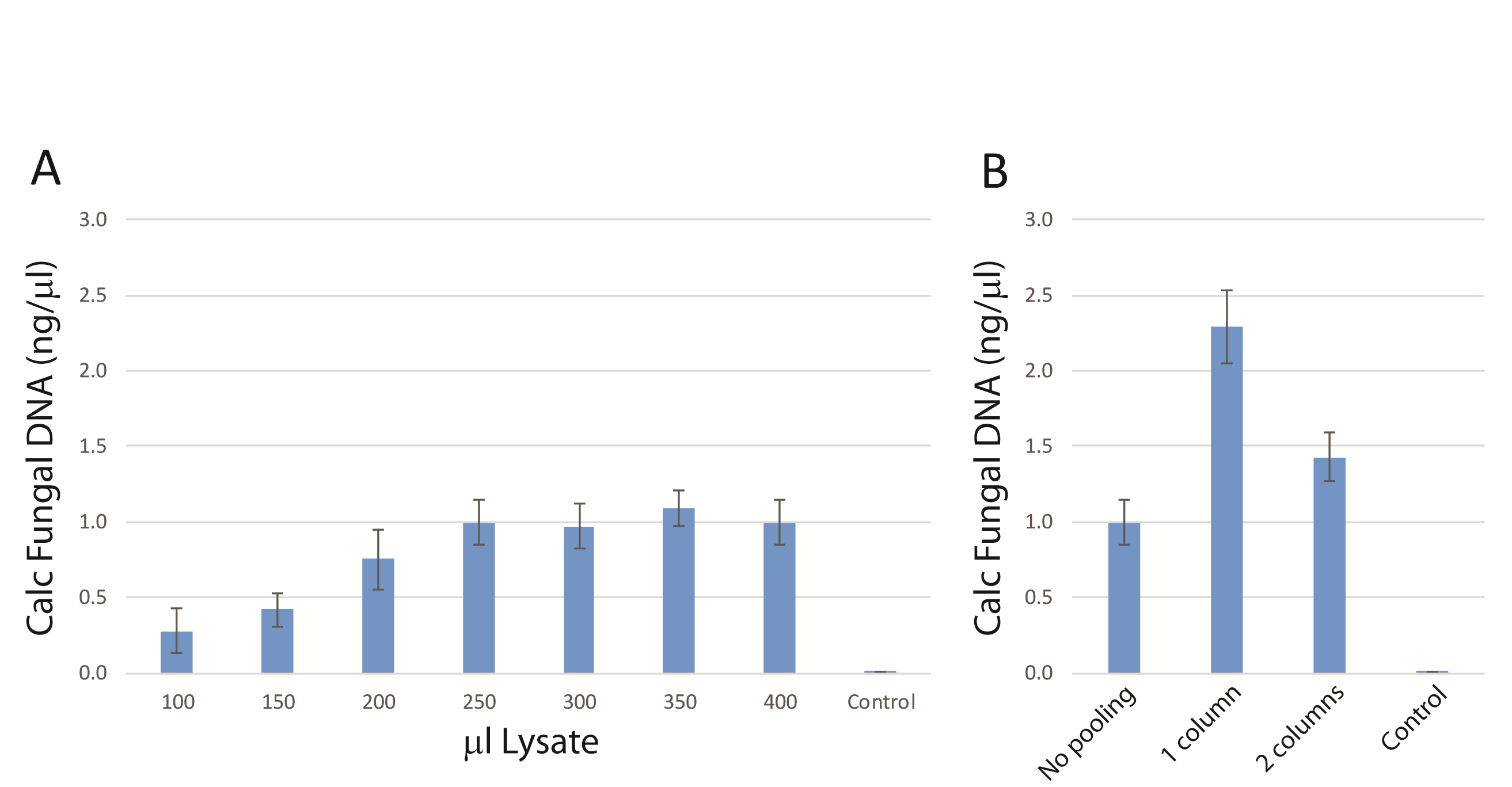

Supplement: S3 Fig — Standardized urine samples from 4 subjects were pelleted and processed using the optimized purification protocol for enzymatic treatment and cell wall disruption. (A) Prior to the addition of proteinase K, varying quantities of the total sample lysate were transferred to new tubes for digestion and DNA column binding. Lysate quantities ≥250 μl provided equivalent yields. (B) Prior to the addition of proteinase K, sample lysates were divided into 250 μl aliquots. Processing of a single 250 μl aliquot (No pooling) was compared to the results if the lysate was split into two aliquots and processed in parallel, then later pooled on either a single DNA-binding column and eluted as a single sample (1 column) or purified separately on two columns, eluted independently and then pooled (2 columns). Control samples were processed in parallel and did not have any input cellular material. (TIF) [file pone.0210306.s003.tif]
